# Associations between food insecurity and Supplemental Nutrition Assistance Program participation with ultra-processed food acquisitions for home consumption in US households

**DOI:** 10.1017/S1368980025101808

**Published:** 2026-01-08

**Authors:** Aarohee Fulay, Filippa Juul, Julia Wolfson, Ana Baylin, Joyce Lee, Euridice Martinez-Steele, Cindy Leung

**Affiliations:** 1 Department of Epidemiology, University of Pittsburgh School of Public Health, Pittsburgh, PA, USA; 2 Center for Epidemiological Studies in Health and Nutrition, University of São Paulo, São Paulo, Brazil; 3 Departments of International Health and Health Policy and Management, Johns Hopkins Bloomberg School of Public Health, Baltimore, MD, USA; 4 Department of Nutritional Sciences, University of Michigan School of Public Health, Ann Arbor, MI, USA; 5 Susan B. Meister Child Health Evaluation and Research Center, Division of Pediatric Endocrinology, University of Michigan Medical School, Ann Arbor, MI, USA; 6 Department of Nutrition, School of Public Health, University of São Paulo, São Paulo, Brazil; 7 Department of Nutrition, Harvard T.H. Chan School of Public Health, Boston, MA, USA

**Keywords:** Food insecurity, SNAP, Ultra-processed foods, Food purchases

## Abstract

**Objective:**

Ultra-processed foods (UPFs) are shown to promote disease. Research shows high UPF intake with food insecurity and SNAP participation. However, no research has quantitatively examined UPF acquisitions (which includes purchases) by food insecurity and SNAP status in US households. This analysis examines food insecurity and SNAP participation with UPF acquisitions for home consumption.

**Design:**

Food insecurity was assessed through the ten-item Adult Food Security Survey. Household SNAP participation was considered affirmative if any member of the household reported receiving SNAP benefits. Household UPF acquisitions/purchases for home consumption (as a percentage of total energy acquired/purchased) were determined by the NOVA classification system. Multivariable linear regressions adjusted for household sociodemographic characteristics quantified associations between food insecurity and SNAP participation with UPF acquisitions for home consumption in US households.

**Setting:**

The USA.

**Participants:**

3949 households from the National Household Food Acquisition and Purchase Survey.

**Results:**

15·5 % and 13·9 % of US households experienced marginal food security and food insecurity, respectively. Adjusted means for UPF acquisition for home consumption across food security and SNAP categories ranged from 53·2 % to 57·0 %. Marginal food security was associated with 3·8 % higher UPF acquisitions for home consumption (*P* = 0·0039) compared with households with high food security. However, there was no association with food insecurity or SNAP.

**Conclusions:**

UPF acquisitions for home consumption were high for US households across food security and SNAP categories. Marginal food security was associated with higher UPF acquisitions for home consumption in US households. However, we observed no associations between food insecurity and SNAP participation with UPF acquisitions. More research on drivers of this association for households with marginal food security should be conducted.

Food insecurity, a state of inconsistent or inadequate access to sufficient food^(1)^, is a global issue – approximately 870 million persons experience food insecurity^(2)^. However, the measurement and severity of food insecurity can vary between and within nations. Subsequently, programmes and policies to address food insecurity and associated diet/health outcomes can vary by nation as well. In the USA, food insecurity impacts approximately one in ten US households^(3)^. In US adults, food insecurity has been associated with poor dietary intake^(4)^, CVD^(5)^ and diabetes^(6)^. In US children and/or adolescents, food insecurity has been associated with higher intake of sugar-sweetened beverages and fast food^(7)^ and prediabetes risk^(8)^.

The Supplemental Nutrition Assistance Program (SNAP) – the largest federal nutrition assistance programme^(9)^ – has been shown to improve food security^(10)^ because it provides benefits to programme participants to purchase additional food^(9)^. However, a cost analysis determined that SNAP benefits are likely not enough to facilitate healthy consumption patterns^(11)^. SNAP participation has been associated with lower quality food purchases^(12)^ and poor dietary intake^(13)^. SNAP participation has also been associated with negative cardiometabolic outcomes^(14)^.

Recent evidence has shown that food insecurity and SNAP participation are associated with higher intake of ultra-processed foods (UPFs) among lower-income US adults^(15)^. Similarly, new research has shown some evidence for an association between food insecurity and higher intake of certain UPF items in US children and/or adolescents^(7,16)^. UPFs – defined as ‘formulations of ingredients, mostly of exclusive industrial use, that result from a series of industrial processes’^(17)^ – are a public health concern because of their low nutritional value^(18)^ and their association with chronic disease^(19,20)^. Excessive consumption of highly processed food is an emerging global issue as well, with high consumption particularly in the USA and the United Kingdom, accounting for more than half of daily caloric intake, on average^(21)^. Typically, UPFs are more affordable^(22)^, with the average UPF costing $0·55 per 100 calories and the average unprocessed food costing $1·45 per 100 calories^(22)^. For example, fruits and vegetables can cost $1·33 and $1·68 per 100 calories, approximately 2–3 times as much as UPFs^(22)^. Moreover, prices for unprocessed foods have risen over the years, while UPF prices have remained more consistent^(22)^.

To date, no research has quantitatively examined the associations between food insecurity and SNAP participation with UPF acquisitions (including purchases), a potential facilitator of high UPF intake. For example, it is unknown if one of the reasons for high UPF intake in lower-income individuals with food insecurity and/or SNAP participation is higher household acquisition of UPFs. Alternatively, UPF acquisition patterns can be similar across households, but whole foods may be consumed less compared with UPFs, thus driving higher *consumption* without higher purchases/acquisitions. This information could enable the development of interventions at appropriate points on the pathways between food insecurity and SNAP participation with higher UPF intake. Finally, this knowledge could inform evidence-based improvements to current nutrition policies and programmes.

Therefore, this paper examines the association between food insecurity and household SNAP participation with household UPF acquisitions (including purchases) for home consumption for 3949 US households using data from the 2012–2013 National Household Food Acquisition and Purchase Survey (FOODAPS)^(23)^. It is hypothesised that both food insecurity and household SNAP participation will be associated with higher household UPF acquisitions for home consumption for US households.

## Methods

### Dataset

The data came from the United States Department of Agriculture (USDA) FOODAPS. This nationally representative, cross-sectional, complex survey was administered in 2012–2013 and contains data from 4826 US households^(23)^. Survey participants are asked to record their food acquisitions and purchases for 7 d. Information is collected on food items, groups of acquisitions/purchases (transactions), individuals and households. Additionally, the survey asks about food security, federal nutrition assistance programme participation, sociodemographic characteristics and more^(23)^. The primary household respondent – the household member most responsible for food shopping and/or meal planning – provided the requested information on behalf of the household^(23)^.

### Sample

The current analytical sample consists of 3949 US households that had non-missing information on food acquisitions/purchases, food insecurity, household SNAP participation and other variables of interest. Households were excluded from the sample if they were missing food-at-home (FAH) data, reported fewer than 6 or greater than 150 FAH items, reported no item data or reported excessive total energy acquired for home consumption (≥ 450 000 kcal across 7 d for the household). This latter criterion excluded one outlier of 2 716 826·75 kcal, which was deemed not plausible even when considering that the largest household size in our sample consists of fourteen individuals, as this number would correspond to 27 722·72 kcal acquired per individual per day in a household of fourteen. Meanwhile, a threshold of 450 000 kcal would correspond to ∼4500 kcal per individual per day in a fourteen-person household, which is plausible, especially since not all food acquired for home consumption is consumed immediately or completely. These criteria were used previously for a similar analysis^(24)^.

### Exposures

The exposure of food insecurity was assessed through the USDA 30-d Adult Food Security Survey Module^(25)^. The primary household respondent answered the ten-item survey. Affirmative responses to 0, 1–2, 3–5 or 6+ questions were coded as high food security, marginal food security, low food security or very low food security, respectively^(23)^. High food security signifies no food security issues. Marginal food security means a few food security issues but without major changes in dietary intake. Low food security indicates a lower quality diet without a major decrease in overall intake. Very low food security means that both diet quality and intake are affected^(1)^. For this analysis, the categories of low and very low food security were combined to create a ‘food insecurity’ category.

The exposure of household SNAP participation was deemed affirmative if one or more household members reported receipt of SNAP benefits at the time of the survey^(23)^. SNAP is for lower-income US households, with current eligibility criteria as 130 % of the federal poverty line or below^(26)^. For a four-person household, this is approximately $40 500^(27)^. This income eligibility applies to the early 2010s as well, where approximately 80 % of SNAP recipient households were approximately 100 % federal poverty line or below^(28)^.

### Outcome

The outcome variable was household UPF acquisitions (including purchases) for home consumption as a percentage of total household energy acquired for home consumption. For simplicity, we refer to UPF acquisitions throughout the paper and include purchases within this label. To calculate this variable, FOODAPS FAH acquisition/purchase data^(23)^ were used to determine the food items acquired for each household. Food items were classified as UPFs or non-UPFs by their food codes or standard reference codes through the NOVA classification system^(17)^. Non-UPFs contain the NOVA categories of processed food, processed culinary ingredients and unprocessed or minimally processed food. Examples of UPFs include sugar-sweetened beverages and cereal bars, while examples of non-UPFs include canned vegetables, cane sugar and sweet potatoes^(17)^. For reference, a table with the NOVA categories, detailed definitions and examples can be found in the supplementary information of this publication^(17)^. Additional information regarding the application of the NOVA classification to FOODAPS data has been published elsewhere^(24)^. The energy (in kilocalories) of the food items was calculated by using the edible weight of the food (in grams) acquired. For relevant food items, values imputed by the Economic Research Service for the weight of food in grams^(23)^ were used. The energy content of the UPF items was aggregated by household to create household UPF kilocalories acquired/purchased for home consumption. This number was then divided by total household energy acquired/purchased for home consumption to generate the outcome variable.

### Covariates

The covariates were age (16–19, 20–35, 36–59 and 60+ years), sex (male/female), race/ethnicity, marital status, education status of the primary household respondent, household income-to-poverty ratio and household size. Race/ethnicity was coded as the following categories: non-Hispanic White, non-Hispanic Black, Hispanic ethnicity and other non-Hispanic race. Marital status was a binary variable of ‘married’ or ‘not married’. The education status variable consisted of two categories: high school graduate or less and more than high school graduate. Household income-to-poverty ratio is a variable calculated by FOODAPS through the division of household income by the federal poverty guideline^(23)^. Finally, household size is the number of non-guest individuals in the household^(23)^.

### Statistical analysis

Weighted means and proportions were calculated for descriptive statistics by food security status. Rao–Scott *χ*^2^ tests and simple linear regressions were conducted for categorical and continuous variables, respectively. Separate multivariable linear regressions evaluated the association between food insecurity and SNAP participation with UPF acquisitions in the main analyses. Model 1 was adjusted for primary household respondent age and sex, household size and total energy acquired/purchased for home consumption. Model 2 was adjusted for all covariates, and the SNAP analyses were further adjusted for food insecurity status. To test for interaction, we included a statistical interaction term of food security status by SNAP status and examined the *P*-value. FOODAPS household weights, strata and clustering^(23)^ were applied for all analyses using survey procedures in SAS Version 9.4 (Cary, NC). An alpha level of 0·05 was used to determine statistical significance.

## Results

**Figure 1.**
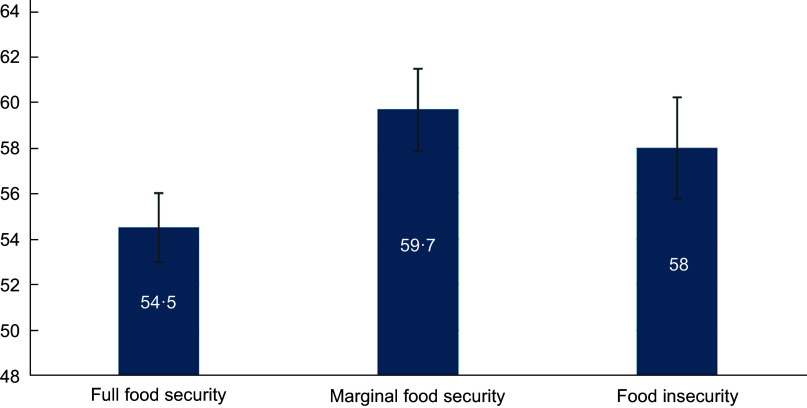
Ultra-processed food acquisition (% total energy) by food security status in the National Food Acquisition and Purchase Survey Sample (*n* 3949 households).

In the sample, 15·5 % of households experienced marginal food security, and 13·9 % experienced food insecurity. UPF acquisitions were 58 % of total energy acquired in households with food insecurity, 59·7 % for marginal food security and 54·5 % for full food security (Figure 1). Food insecurity was associated with lower household respondent age (*P* < 0·0001), education (*P* < 0·0001) and household income-to-poverty ratio (*P* < 0·0001). Household respondent race/ethnicity varied by food security status (*P* < 0·0001) such that households with marginal food security and households with food insecurity were more likely than a household with full food security to have a household respondent who identified as non-Hispanic Black, Hispanic ethnicity or other non-Hispanic race. Food insecurity was also associated with ‘not married’ primary household respondent status (*P* < 0·0001), larger household size (*P* < 0·0001) and household SNAP participation (*P* < 0·0001) (Table 1).

**Table 1.** Associations between food insecurity and sociodemographic characteristics in US households (*n* 3949) from FOODAPS 2012–2013

	Overall sample weighted %	*n*	Full food security weighted %	*n*	Marginal food security weighted %	*n*	Food insecurity weighted %	*n*	*P*-value
Overall (%)	100·0	3949	70·6	2103	15·5	808	13·9	1038	
Household respondent age (%)									**< 0·0001**
16–19 years	0·4	38	0·4	22	0·3	4	0·6	12	
20–35 years	21·6	1166	19·5	550	25·6	274	27·7	342	
36–59 years	47·0	1849	45·3	919	48·9	375	53·2	555	
60+ years	31·0	896	34·8	612	25·2	155	18·5	129	
Male household respondent (%)	29·9	968	31·0	554	26·1	173	28·1	241	0·1981
Household respondent race/ethnicity (%)									**< 0·0001**
Non-Hispanic White	70·3	2392	77·8	1463	55·1	409	49·3	520	
Non-Hispanic Black	9·9	463	7·1	183	15·9	133	17·6	147	
Hispanic ethnicity	12·9	795	8·5	300	21·9	202	25·5	293	
Other non-Hispanic race	6·8	299	6·6	157	7·1	64	7·7	78	
Household respondent education > high school graduate (%)	66·5	2166	72·8	1328	57·0	401	45·2	437	**< 0·0001**
Household respondent married (%)	48·0	1808	53·2	1124	38·9	321	31·8	363	**< 0·0001**
Household SNAP participation (%)	11·5	1149	5·6	351	16·6	291	35·6	507	**< 0·0001**
	Overall sample weighted mean	Weighted se	Full food security weighted mean	Weighted se	Marginal food security weighted mean	Weighted se	Food insecurity weighted mean	Weighted se	*P*-value
Household income-to-poverty ratio, mean (se)	375·0	12·8	440·0	14·3	263·6	12·0	169·8	8·0	**< 0·0001**
Household size, mean (se)	2·5	0·05	2·4	0·04	2·8	0·11	2·7	0·09	**< 0·0001**

FOODAPS, National Household Food Acquisition and Purchase Survey; SNAP, Supplemental Nutrition Assistance Program.

Statistically significant estimates at alpha = 0·05 are bolded.

All analyses used survey procedures that accounted for the complex survey design strata, clusters and weights.

Simple linear regressions were used for continuous variables, and Rao–Scott *χ*^2^ tests were used for categorical variables.

Across food security and SNAP participation levels, fully adjusted mean UPF acquisitions for home consumption as a percentage of total energy acquired for US households ranged from 53·2% to 57·0%. In the Model 1 food insecurity analysis, marginal food security was associated with 4·6 % higher household UPF acquisition for home consumption as a percentage of total energy acquired (*P* = 0·0003). There was also an association between food insecurity and 3·0 % higher UPF acquisition (*P* = 0·0282). The *P*-for-trend for this association was statistically significant (*P* = 0·0036). After multivariable adjustment, marginal food security was associated with a 3·8% higher UPF acquisition (*P* = 0·0039), and the association with food insecurity was attenuated (*P* = 0·1812). The *P*-for-trend for this association was marginally statistically significant (*P* = 0·0689) (Table 2).

**Table 2.** Associations between food insecurity and household ultra-processed food acquired (as a percentage of total energy acquired) for home consumption in US households (*n* 3949) from FOODAPS 2012–2013

	Model 1				Model 2			
	Adjusted mean	Estimate	95 % CI	*P*-value	Adjusted mean	Estimate	95 % CI	*P*-value
			LL, UL				LL, UL	
Full food security	55·4	Ref.			53·2	Ref.		
Marginal food security	60·0	4·6	2·2, 6·9	**0·0003**	57·0	3·8	1·3, 6·4	**0·0039**
Food insecurity	58·4	3·0	0·3, 5·6	**0·0282**	55·3	2·1	−1·0, 5·3	0·1812
*P*-for-trend				**0·0036**				0·0689

FOODAPS, National Household Food Acquisition and Purchase Survey; LL, lower limit; UL, upper limit.

Statistically significant estimates at alpha = 0·05 are bolded.

All analyses used survey procedures that accounted for the complex survey design strata, clusters and weights.

Multivariable linear regressions were used to calculate beta coefficient estimates and adjusted means.

Model 1 is adjusted for household respondent age, household respondent sex, household size and total energy acquired/purchased.

Model 2 is adjusted for Model 1 + household respondent race/ethnicity, education, marital status and household income (as a quadratic expression).

The Model 1 SNAP analysis showed an association between household SNAP participation and 3·8 % higher UPF acquisition (*P* = 0·0019); however, after multivariable adjustment, the association was NS (*P* = 0·3164) (Table 3). The interaction between SNAP and food security status was not statistically significant (*P* = 0·4733).

**Table 3.** Associations between SNAP participation and household ultra-processed food acquired (as a percentage of total energy acquired) for home consumption in US households (*n* 3949) from FOODAPS 2012–2013

	Model 1				Model 2			
	Adjusted mean	Estimate	95 % CI	*P*-value	Adjusted mean	Estimate	95 % CI	*P*-value
			LL, UL				LL, UL	
SNAP non-participation	55·9	Ref.			54·9	Ref.		
SNAP participation	59·8	3·8	1·5, 6·1	**0·0019**	56·4	1·5	−1·5, 4·5	0·3164

FOODAPS, National Household Food Acquisition and Purchase Survey; LL, lower limit; SNAP, Supplemental Nutrition Assistance Program; UL, upper limit.

Statistically significant estimates at alpha = 0·05 are bolded.

All analyses used survey procedures that accounted for the complex survey design strata, clusters and weights.

Multivariable linear regressions were used to calculate beta coefficient estimates and adjusted means.

Model 1 is adjusted for household respondent age, household respondent sex, household size and total energy acquired/purchased.

Model 2 is adjusted for Model 1 + household respondent race/ethnicity, education, marital status, household income (as a quadratic expression) and food insecurity status.

## Discussion

Within this national sample of US households, mean household UPF acquisitions intended for home consumption were high among all households, regardless of food security status and household SNAP participation. For all food security categories, UPF acquisition accounted for more than half of the energy acquired for home consumption and was especially high for those with marginal food security. Marginal food security was associated with higher UPF acquisitions for home consumption. On the other hand, food insecurity was not associated with UPF acquisitions. SNAP participation was also not associated with UPF acquisitions. Research shows that UPF consumption in the US is higher than UPF intake in some other industrialised nations – for example, Italy, which has UPF intakes approximately 13–14 %^(29)^ – and comparable to others^(30)^.

This paper is the first to quantitatively examine the associations between food insecurity and SNAP participation with UPF acquisitions for home consumption in US households. Few studies have examined the association between food insecurity and the nutritional quality of food acquired. Gregory *et al*. used FOODAPS data to determine that households with food insecurity were likely to purchase more refined grains and less fruit, dairy products and protein. However, this analysis was conducted in low-income households only, which could be one explanation why our results differ^(31)^. For households with children, reduced food security is associated with a home food environment with more microwaveable or convenience frozen food items^(32)^, which are likely to be classified as UPFs. Finally, Vadiveloo *et al*. found that the nutritional quality of dietary acquisitions for home consumption was lower in households that participated in SNAP and experienced food insecurity^(33)^. These results somewhat align with our finding that US households with marginal food security are more likely to acquire UPFs for home consumption. Initially, we found an association between food insecurity and household UPF acquisitions for home consumption, but this association attenuated to non-significance after full multivariable adjustment. Additionally, it is possible that results vary slightly between studies because different nutrition metrics are used – for example, nutrients, food groups or UPF acquisition/purchase.

It is possible that households with marginal food security acquire more UPFs for home consumption for financial reasons. As previously mentioned, a main draw for UPF acquisition is likely the lower cost^(22)^. While there is little evidence on food purchasing patterns specifically based on food security status, households that are marginally food secure may acquire UPFs as a more affordable food option^(22)^ in an effort to extend their food budget and avoid food insecurity. Qualitative interviews with marginally food secure households about their purchasing patterns could examine this hypothesis. In fact, research should investigate whether these households utilise the acquisition of cheaper UPFs to fend off food insecurity. Meanwhile, households with food insecurity did not have higher acquisition of UPFs for home consumption in the present study. If future research shows that UPF acquisition for home consumption is a strategy to prevent households from falling into food insecurity, then, by comparison, it would be expected that households with food insecurity would not have higher UPF acquisition.

Meanwhile, ample literature on the association between SNAP participation and food acquisitions exists. One study found that SNAP participation is associated with acquisitions of lower nutritional quality^(34)^, and another found that it was associated with higher purchases of sugar-sweetened beverages, processed meat and sweeteners and toppings with lower purchases of fruit and salty snacks^(12)^. Another report found no major differences in food item purchases between SNAP and non-SNAP households, with the exception of soft drinks, of which SNAP households purchased slightly more^(35)^. Meanwhile, Chen *et al*. used FOODAPS data to examine food purchased for home consumption in low-income households and found that SNAP participation was associated with less healthy purchases for households that were deemed ‘less nutrition-oriented’ but not for households that were deemed ‘nutrition-oriented’^(36)^. Using FOODAPS data, Basu *et al*. observed that SNAP participation was associated with better quality acquisitions in counties with high cost-of-living^(37)^. In general, it seems that the evidence for SNAP is somewhat mixed and that associations may vary based on household characteristics and local factors. In our study, the link between SNAP and UPF acquisition for home consumption disappeared after multivariable adjustment. The adjustment for food security status – included in the model as a potential confounder – may have played a role in this attenuation. Additionally, future research should examine if UPF acquisition outside of the home may drive higher UPF intake in SNAP participants^(15)^ rather than home acquisitions.

Nonetheless, an association between marginal food security and higher UPF acquisition is concerning. Higher UPF acquisition for home consumption in households with marginal food security likely contributes to UPF intake in adults with marginal food security. High UPF intake is detrimental to health due to its long-term association with CVD^(19)^ and diabetes^(20)^. Additionally, provided the potentially addictive qualities of UPFs^(38)^ and association with binge eating^(39)^, the inequitable exposure of marginally food secure households to this class of foods is particularly troubling.

In addition to examining the association between marginal food security and higher household UPF acquisition for home consumption, it is important to examine the other study findings within the broader backdrop of literature on UPF intake, as some key distinctions appear. In previous research, very low food security (when compared with full food security) was associated with 3·1 % higher UPF intake, and SNAP participation (when compared with income-eligible non-participation) was associated with 1·7 % higher UPF intake in lower-income US adults^(15)^. Therefore, while we found that marginally food secure households are more likely to acquire UPFs for home consumption, lower-income adults with food insecurity are more likely to consume UPFs^(15)^. Additionally, while we found no associations for household SNAP participation and UPF acquisition, lower-income adults from households that participate in SNAP were more likely to consume UPFs^(15)^.

The differential findings between UPF acquisition and intake raise some important considerations. To begin, it points to the difference between acquisition and consumption. Generally, the acquisition of a food item may not correlate exactly with consumption. While fresh foods can go to waste and thus be consumed less, UPFs are generally shelf-stable, which enables them to persist as a food option even when other food supplies may run low, and thus be consumed more. SNAP participants have reported a preference for UPFs for their shelf-stable qualities^(40)^. SNAP participants also tend to select UPFs because they require minimal preparation and household children are likely to consume them (and decrease the likelihood of food waste)^(40)^. It is possible that individuals with food insecurity choose UPFs for similar reasons but that has not been examined in the literature – however, a qualitative study did indicate that some SNAP participants would buy and store UPFs in an attempt to reduce the risk of food insecurity later in the month^(40)^. Therefore, though food insecurity and SNAP participation are not associated with higher UPF acquisition for home consumption, disproportionately high consumption of available UPFs could occur in lower-income US adults with food insecurity or SNAP participation due to long shelf-life, lower preparation requirements or other reasons. Overall, factors such as constraints within the home (such as time or money) or food acquired outside the home may drive high UPF intake in lower-income US adults with food insecurity or SNAP participation. The results also show that all households could benefit from a reduction in UPF acquisition for home consumption, especially households with marginal food security.

Consequently, future research should examine mechanisms for higher UPF acquisition and intake in lower-income populations that have less food security or participate in SNAP. For example, qualitative work should be conducted with marginally food secure households to see what drives higher UPF acquisition for home consumption in that group (and if it becomes a coping strategy to maintain food security status). Focus groups for individuals who are food insecure could examine reasons for higher UPF intake in this population. Moran *et al*. have examined drivers of UPF purchases and intake in SNAP households with children^(40)^, and this work could be expanded to include all SNAP participating households. A quantitative analysis on the association between food insecurity and SNAP participation with UPF acquisition for consumption outside the home (such as fast food places, restaurants, etc.) would also shed light on this issue. Understanding these mechanisms could provide the basis for critical public health interventions. Research has shown that product placement and promotion are associated with purchases^(41)^; therefore, perhaps local government and non-profits could provide incentives for supermarkets and grocery stores to promote non-UPF items. Higher SNAP benefits may facilitate acquisitions of non-UPF food items, such as fresh fruits/vegetables, as a main reason for high UPF acquisition in this group may be the more affordable cost^(22)^. In fact, a recent review found that fruit/vegetable incentives – which augment existing SNAP benefits – have enabled SNAP participants to acquire and consume more fruits/vegetables^(42)^. Higher SNAP benefits may help overcome the hurdle of current income limitations, which are likely exacerbating UPF acquisitions. Finally, broader social and economic policies that improve finances and time scarcity^(43)^ would likely improve a lower-income household’s ability to acquire unprocessed foods and prepare more nutritious meals (as well as decrease fast food consumption).

This study has multiple strengths. To start, it uses national data. Also, the FAH acquisition/purchase data may be less susceptible to recall bias than dietary intake data due to additional reliance on receipts and barcodes^(23)^. One of the main limitations of the analysis is the cross-sectional data collection, which can lessen the ability to infer causality. However, acquisitions are likely a result of food security status and/or household SNAP participation rather than the opposite, so reverse causality is relatively unlikely. As always, reporting bias may be an issue for variables such as food security status, household SNAP participation and income, due to their private nature^(44)^. However, FOODAPS takes measures to account for non-response bias, and methods are used to obtain the most accurate data possible^(23)^. Additionally, we restricted our sample based on data availability/plausibility, and therefore, our analytic sample may differ slightly from the broader study sample. Another limitation is that the food security status of the household respondent was utilised as a proxy measure for that of the household. Furthermore, the 30-d measure might not be representative of long-term food security status or sporadic periods of food insecurity. However, FOODAPS does not provide a 12-month measure of household food security^(23)^, so the best available measure was used. An additional limitation is the timing of the data collection. While this dataset is unique in its examination of food acquisition data at the national level, the data were collected from 2012 to 2013. The prevalence of food insecurity in 2012 was 14·5 %^(45)^ and in 2023 was 13·5 %^(46)^ and the number of Americans on SNAP was 44·7 million in 2011^(47)^ and 42·1 million in 2023^(48)^. Meanwhile, recent estimates show that UPF intake in 2011–2012 is comparable to UPF intake in 2017–2018^(49)^. Therefore, based on recent data, food insecurity, SNAP participation and UPF intake rates have been similar over recent years. However, these trends may shift moving forward, so newer data should be collected and utilised to further examine these associations in the present day. Overall, the relevance of this research question persists with time. Finally, the possibility of residual confounding cannot be excluded. Even with the limitations, this paper addresses a critical gap in the evidence base and is the first to quantitatively examine UPF acquisition for home consumption in households with decreased food security and households that participate in SNAP.

To conclude, household UPF acquisition for home consumption was high for US households with varying levels of food security and SNAP participation status. Marginal food security was associated with higher levels of UPF acquisition for home consumption; these high levels of UPFs in the home environment very likely contribute to UPF intake in this population, which is concerning due to the many negative health effects of UPFs^(19,20,50)^. Research on the driving factors creating high acquisition of UPFs among US households should be examined to inform evidence-based interventions to shift food acquisition for home consumption towards less processed foods. Although food insecurity and household SNAP participation are associated with higher UPF intake in lower-income US adults, there was no evidence that food insecurity or household SNAP participation are associated with higher UPF acquisition for home consumption. Therefore, research should be conducted to assess alternative drivers of high UPF intake in these groups, such as UPF acquisition outside the home, a reliance on UPFs when other food options run low or limitations around meal preparation feasibility. UPF acquisitions for home consumption may be an important leverage point for improving the home food environment for households with marginal food security.
